# The SbbHLH041–*SbEXPA11* Module Enhances Cadmium Accumulation and Rescues Biomass by Increasing Photosynthetic Efficiency in Sorghum

**DOI:** 10.3390/ijms241713061

**Published:** 2023-08-22

**Authors:** Huinan Wang, Junxing Yu, Bin Zhu, Lei Gu, Hongcheng Wang, Xuye Du, Tuo Zeng, Heng Tang

**Affiliations:** 1 School of Life Sciences, Guizhou Normal University, Guiyang 550025, China; huinanwang123@163.com (H.W.); yujunxing00@163.com (J.Y.); zhugg130@126.com (B.Z.); 201808009@gznu.edu.cn (L.G.); wanghc@gznu.edu.cn (H.W.); duxuye@gznu.edu.cn (X.D.); 2 National Key Laboratory of Wheat Breeding, Agronomy College, Shandong Agricultural University, Tai’an 271002, China

**Keywords:** sorghum, expansin 11, Cd accumulation, SbbHLH041, transcriptional regulation

## Abstract

In plants, expansin genes are responsive to heavy metal exposure. To study the bioremediary potential of this important gene family, we discovered a root-expressed expansin gene in sorghum, *SbEXPA11*, which is notably upregulated following cadmium (Cd) exposure. However, the mechanism underlying the Cd detoxification and accumulation mediated by *SbEXPA11* in sorghum remains unclear. We overexpressed *SbEXPA11* in sorghum and compared wild-type (WT) and *SbEXPA11*-overexpressing transgenic sorghum in terms of Cd accumulation and physiological indices following Cd. Compared with the WT, we found that *SbEXPA11* mediates Cd tolerance by exerting reactive oxygen species (ROS)-scavenging effects through upregulating the expression of antioxidant enzymes. Moreover, the overexpression of *SbEXPA11* rescued biomass production by increasing the photosynthetic efficiency of transgenic plants. In the pot experiment with a dosage of 10 mg/kg Cd, transgenic sorghum plants demonstrated higher efficacy in reducing the Cd content of the soil (8.62 mg/kg) compared to WT sorghum plants (9.51 mg/kg). Subsequent analysis revealed that the SbbHLH041 transcription factor has the ability to induce *SbEXPA11* expression through interacting with the E-box located within the *SbEXPA11* promoter. These findings suggest that the SbbHLH041–*SbEXPA11* cascade module may be beneficial for the development of phytoremediary sorghum varieties.

## 1. Introduction

Abiotic stressors, including heavy metals, nutrient deficiencies, extreme temperature, salinity/alkalinity, and drought, can negatively impact plant health and productivity [1]. In recent decades, there has been a notable global escalation in soil heavy metal pollution due to extensive industrial and anthropogenic activities. Furthermore, heavy-metal-contaminated soils act as sources of food chain contamination, resulting in detrimental effects on human, animal, and plant health [2]. Cadmium (Cd) is a nonessential nutrient and is harmful to plant cells [3]. For instance, Cd can damage the electron transport chain, generating reactive oxygen species (ROS) and disrupting photosynthetic processes [4]. Additionally, Cd inhibits the activities of key enzymes and the acquisition of essential nutrients, reducing both the quality and yield of crop plants [5,6]. Several methods have been proposed to remove heavy metal ions from soil [6,7], including phytoremediation, physical remediation, chemical remediation, and bioremediation [1,2,3,4]. Physical and chemical remediation methods provide quick remediation but are costly and cause secondary pollutants [2]. Phytoremediation is an economical and environmentally–sustainable technique for decontaminating Cd-polluted soils [7]. Sorghum (*Sorghum bicolor* L.) is a multipurpose plant grown for grain, forage, sugar, lignocellulosic bioenergy, and phytoremediation. Therefore, it is necessary to identify Cd-stress-related genes in order to bioengineer Cd-tolerant sorghum varieties for the purpose of phytoremediation.

Expansin proteins are essential to the processes of cell elongation and division, as well as multiple abiotic stress responses [8]. Expansins are common in plants and are categorized into four subfamilies: expansin-like B (EXLB), expansin-like A (EXLA), β-expansin (EXPB), and α-expansin (EXPA) [9]. The extensive expansin gene family, containing numerous expansin and expansin-like genes, has been studied in an array of plants. For instance, in Arabidopsis, *AtEXP18* and *AtEXP7* are involved in the formation of root hairs [10]. Likewise, *HvEXPB1* mediates the inception of root hair development [11]. Still other expansins have been found to be abiotic-stress-responsive [12]. The Tibetan wild barley gene *HvEXPB7* promotes the elongation of root hairs, thus conferring enhanced drought tolerance [13]. In transgenic Arabidopsis, the overexpression of *TaEXPA2* enhances salinity resistance [14]. Notably, overexpression of the *PttEXPA8* gene from *Populus tomentosa* confers resistance to Cd exposure, salinity, cold temperature, and drought [15]. Other expansins are also involved in heavy metal stress, particularly Cd stress. For instance, the poplar expansin gene *PtoEXPA12* was shown to enhance the Cd content in transgenic tobacco [16], and the common wheat expansin gene *TaEXPA2* was found to enhance Cd-resistance [17]. Based on these results, expansins appear to be a valuable resource for developing crops resistant to Cd toxicity.

In plants, transcription factors (TFs) play key roles in transcriptionally regulating the expression of stress-responsive genes. TFs can act cooperatively or independently to control gene expression [18]. TF proteins bind to specific cis-acting elements present in target gene promoters, functioning to activate or inhibit gene expression [19], and contributing to the formation of complex regulatory networks. Several TF families regulate diverse abiotic stressors in plants, including the AP2/ERF [20,21], MYB [22,23], WRKY [24,25], bHLH [26], NAC [27], and bZIP [28,29] families. Among these, basic helix–loop–helix (bHLH) TFs are particularly crucial for regulating plant response to heavy metal exposure. For instance, the bHLH TF *AtbHLH29* induces the expression of the iron (Fe)-accretion genes *IRT1* and *FRO2* by interacting with either *AtbHLH39* or *AtbHLH38* [30]. In Arabidopsis, the co-overexpression of *AtbHLH29* and *AtbHLH38* constitutively activates the expression of *HMA3* and *IREG2*, which play key roles in heavy metal detoxification [31]. *AtbHLH38* and *AtbHLH101* expression is mediated by the MYB49 TF, leading to activation of the Cd transporter gene *IRON-REGULATED TRANSPORTER1* [32]. Finally, *ZmbHLH105* increases resistance to manganese (Mn) by regulating the antioxidative scavenging of ROS and the expression of Mn and Fe transporters [33].

Previous studies have successfully identified a number of Cd-stress-responsive genes and validated their functions in plants [20,21,22,23,24,25,26,27]. However, these transgenic plants are not suitable for phytoremediation purposes due to the limited biomass and lengthy growth period of transgenic recipients [13,14,15,16,17]. On the other hand, sorghum, as a C4 plant, exhibits substantial biomass, a shorter growth period, and simple cultivation methods. Consequently, sorghum holds distinct advantages over other plants for soil remediation purposes. There is little research into the roles played by *expansins* in Cd stress tolerance in sorghum, and even less is known about their transcriptional regulatory network in this important crop. Here, we discovered that *SbEXPA11* expression was notably induced by Cd exposure. Further analysis indicated that the SbbHLH041 TF could directly activate SbEXPA11 expression by binding to its promoter. Subsequently, we generated transgenic sorghum plants overexpressing *SbEXPA11* and found that *SbEXPA11*-overexpressing seedlings were highly tolerant of Cd, with high Cd accumulation and biomass production under Cd stress. Moreover, the transgenic sorghum was found to reduce Cd residues in the soil. The objective of this study is to investigate the regulatory pathway of sorghum *EXPA11* expression and its role in Cd uptake. Additionally, the work aims to develop novel sorghum germplasms that can be utilized for soil remediation, thereby offering new resources for phytoremediation.

## 2. Results

### 2.1. Cloning and Expression of SbEXPA11 in Sorghum

**Figure 1:**
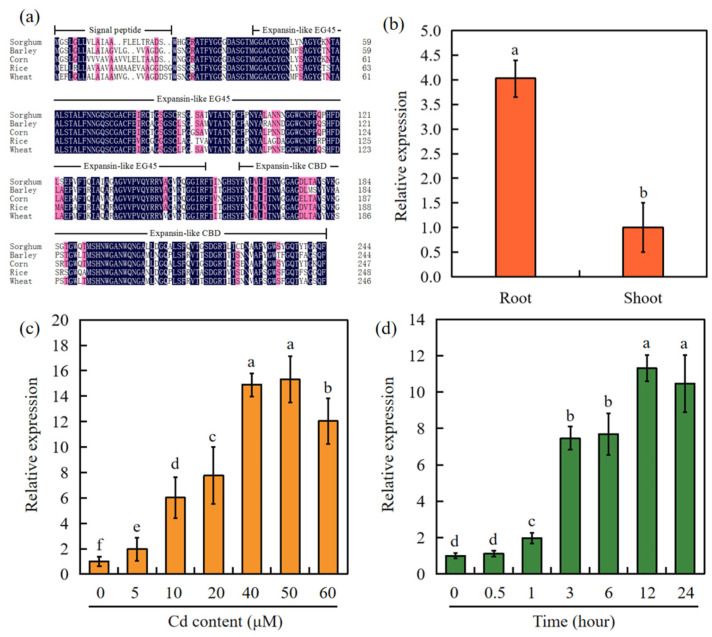
Phylogenetic analysis and expression of SbEXPA11. (**a**) Alignment of the amino acid sequences of EXPA11 of sorghum, barley, corn, rice, and wheat. Expansin-like EG45: expansin, family 45 endoglucanase-like domain; expansin-like CBD: expansin, cellulose-binding-like domain. Non-conservative amino acid sites are marked in red, and conservative regions are marked in blue. (**b**) Under control conditions (0 mM Cd), the expression level of SbEXPA11 in root and shoot. (**c**) Expression analysis of SbEXPA11 under different Cd concentrations. (**d**) Expression analysis of *SbEXPA11* under 50 μM Cd at different times. Error bars are ±SE of *n* = 3. Significant differences analysis was carried out one-way ANOVA and Tukey’s post hoc test. Different letters above the column indicate significant differences (*p* < 0.05).

*SbEXPA11* was notably upregulated in Cd-treated sorghum [34]. However, its phytoremediary potential is unclear. *SbEXPA11* was found to be 735 bp in length and encode a 244 aa polypeptide. The multiple sequence alignment of EXPA11 proteins from sorghum, barley, corn, rice, and wheat revealed that these protein sequences are generally similar, with the exception of the highly variable N-terminal signal peptide region. However, each of the EXPA11 proteins shared highly similar expansin-like EG45 and expansin-like CBD domains (Figure 1a).

We next analysed whether *SbEXPA11* responds to Cd stress. Through RT-qPCR, we found that *SbEXPA11* expression was highest in the roots (Figure 1b). Furthermore, *SbEXPA11* expression was elevated after exposure to different concentrations of Cd, with 50 μM Cd resulting in the highest expression (Figure 1c), particularly 12 h after treatment (Figure 1d).

### 2.2. The SbbHLH041 TF Upregulates SbEXPA11 Expression by Binding Directly to Its Promoter

**Figure 2:**
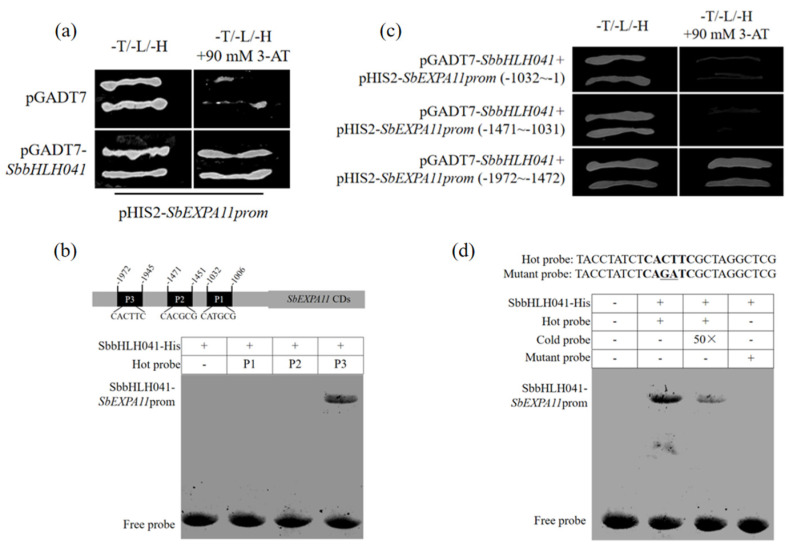
Transcription factor SbbHLH041 binds to *SbEXPA11*-promoter. (**a**) Yeast one-hybrid (Y1H) analysis of the binding of SbbHLH041 to *SbEXPA11*-promoter (*SbEXPA11prom*). (**b**) EMSA showing the binding of SbbHLH041 to the −1972~−1945 region of *SbEXPA11prom*. (**c**) Y1H analysis of the binding of SbbHLH041 to the −1972~−1945 region of *SbEXPA11prom*. (**d**) EMSA showing the binding of SbbHLH041 to the E-box (bold) of *SbEXPA11prom* at −1972~−1945 region. Mutated bases are underlined.

bHLH-type TFs contain a special structural domain (helix–loop–helix (HLH)) which can recognize and bind to the E-box *cis*-elements present in target gene promoters [35]. Here, we obtained a bHLH TF, SbbHLH041, through an Y1H assay using the *SbEXPA11*-promoter as bait (Figure 2a). To further investigate the binding region, the *SbEXPA11*-promoter was divided into three fragments for EMSA. The SbbHLH041 protein was observed to directly bind to the E-box in a P3 fragment (−1972~−1945) of the SbEXPA11-promoter (Figure 2b). Subsequently, Y1H assays demonstrated that only the construct containing the full sequences of pGADT7–*SbbHLH041* and pHIS2–*SbEXPA11prom* (−1972~−1945) was able to survive on SD/–His/–Leu/–Trp medium containing 90 mM 3-AT (Figure 2c). Moreover, mutant P3 fragments in which the CACTTC E-box was present were also generated (Figure 2d). Subsequent EMSA analysis revealed that the mutated DNA probe no longer exhibited binding affinity towards *SbbHLH041* (Figure 2d). These results indicate that SbbHLH041 transcriptionally activates *SbEXPA11* by binding to the E-box of its promoter.

### 2.3. Generation and Identification of SbEXPA11-Overexpressing Sorghum Lines

We generated transgenic sorghum plants overexpressing SbEXPA11 for functional characterization. Following PCR screening, positive transgenic sorghum lines were identified (Supplementary Figure S1a). We then confirmed gene expression in the transgenic plants by RT-qPCR (Supplementary Figure S1b). Three independent T3 transgenic lines, with OE1 and OE2 plants displaying the highest levels of SbEXPA11, were used for follow-up experiments.

### 2.4. Overexpression of SbEXPA11 Increases Cd Uptake and Long-Distance Transport

**Figure 3:**
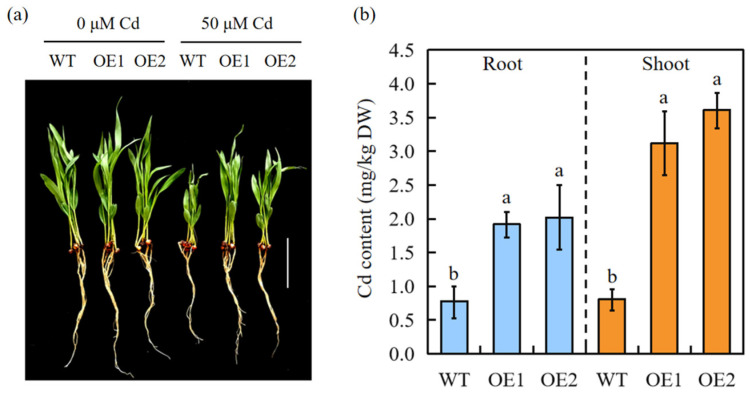
SbEXPA11 transgenic sorghum plants. (**a**) Phenotype of WT and SbEXPA11 transgenic plants under hydroponic conditions. Bar = 1 cm. (**b**) Cd content in shoots and roots of WT and SbEXPA11 transgenic plants under hydroponic conditions. Error bars are ±SE of *n* = 3. Significant differences analysis was carried out by one-way ANOVA and Tukey’s post hoc test. Different letters above the column indicate significant differences (*p* < 0.05).

Both WT and transgenic sorghum plants exhibited severe Cd sensitivity when cultured on a nutrient solution containing Cd. However, the transgenic plants were less affected by Cd stress (Figure 3a). The Cd concentration in the roots and shoots was measured after treatment with 50 μM Cd for 7 days. Transgenic sorghum accumulated significantly more Cd than WT plants (Figure 3b), indicating that SbEXPA11 participates in Cd uptake and transport.

### 2.5. Overexpression of SbEXPA11 Reduces Cd Damage in Transgenic Sorghum

**Figure 4:**
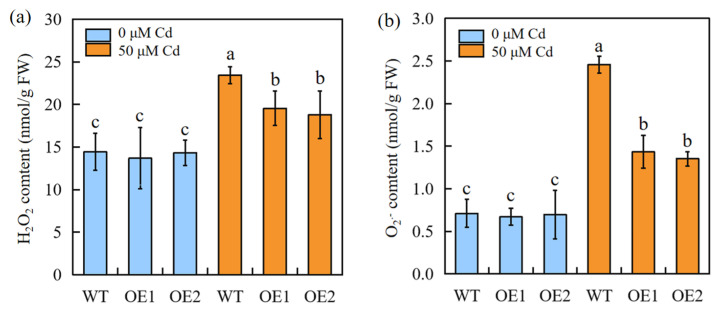
The ROS accumulation of WT and SbEXPA11 transgenic sorghum plants. (**a**) H_2_O_2_ content, (**b**) O_2_^−^ content. WT and SbEXPA11 transgenic plants with or without Cd treatment under hydroponic conditions. Error bars are ±SE of *n* = 3. Significant differences analysis was carried out by one-way ANOVA and Tukey’s post hoc test. Different letters above the column indicate significant differences (*p* < 0.05).

Exposure to Cd stress results in different degrees of oxidative stress in plants. The H_2_O_2_ and O_2_^−^ contents were compared between the WT and transgenic sorghum plants. The results showed that there is no difference in H_2_O_2_ and O_2_^−^ contents between transgenic plants and the WT under normal condition; under Cd stress conditions, for both the WT and transgenic sorghum, the Cd treatment induced a significant H_2_O_2_ and O_2_^−^ burst. Moreover, the transgenic plants accumulated less H_2_O_2_ and O_2_^−^ than the WT plants (Figure 4).

**Figure 5:**
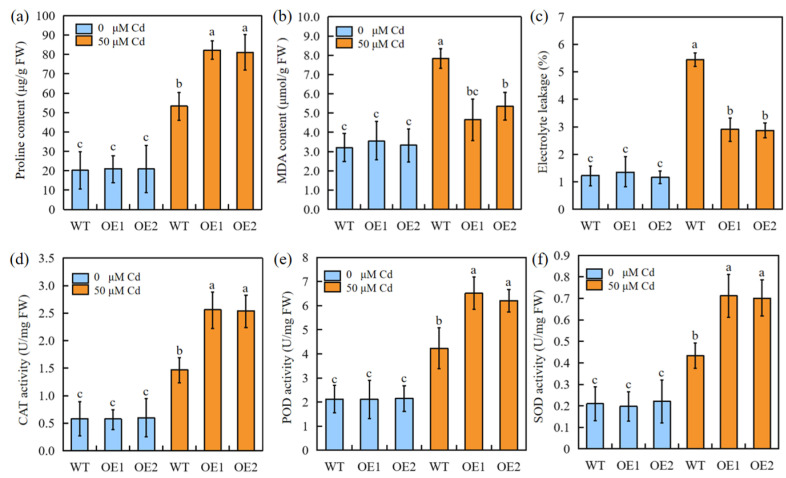
The physiological indicators of SbEXPA11 transgenic sorghum plants. (**a**) Proline content, (**b**) MDA content, (**c**) electrolyte leakage, activities of (**d**) CAT, (**e**) POD, and (**f**) SOD of WT and SbEXPA11 transgenic plants with or without Cd treatment under hydroponic conditions. Error bars are ±SE of *n* = 3. Significant differences analysis was carried out by one-way ANOVA and Tukey’s post hoc test. Different letters above the column indicate significant differences (*p* < 0.05).

Under 50 μM Cd stress, the overexpression of SbEXPA11 was found to reduce Cd damage, as evidenced by favourable changes in proline content and antioxidant enzyme (POD, CAT, and SOD) activity (Figure 5a,d–f). In addition, both electrolyte leakage (%) and MDA content were significantly diminished in transgenic plants (Figure 5b,c). These results suggest that the transgenic sorghum suffered less Cd-induced membrane damage through enhanced ROS scavenging.

### 2.6. Overexpression of SbEXPA11 Increases Phytoremediation Capacity

**Figure 6:**
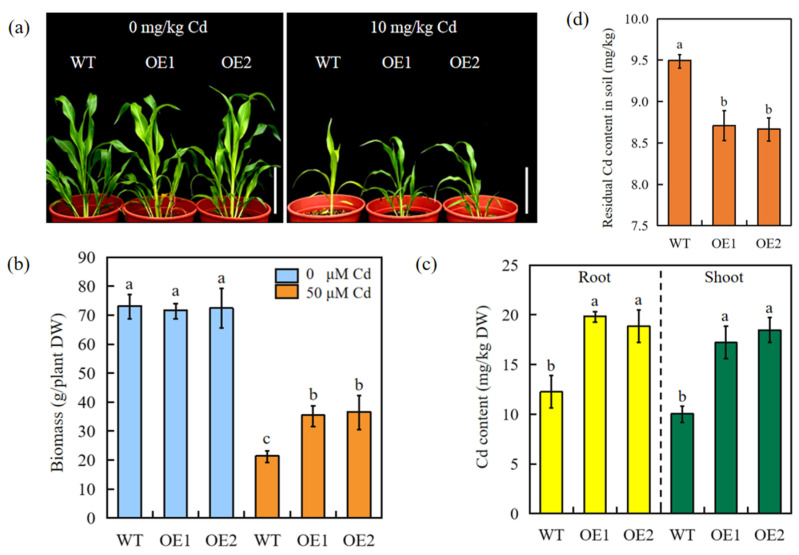
The phenotype of SbEXPA11 transgenic sorghum plants. (**a**) Photograph showing phenotype of WT and *SbEXPA11*-overexpression sorghum seedlings under normal and Cd stress conditions. Scale bar = 10 cm. (**b**) Biomass of WT and *SbEXPA11*-overexpression sorghum with or without Cd treatment under pot experiment. (**c**) Cd content in shoots and roots of WT and SbEXPA11 transgenic plants under pot experiment. (**d**) Residual Cd content in the soil after growing of WT and *SbEXPA11* transgenic plants. Error bars are ±SE of *n* = 3. Significant differences analysis was carried out by one-way ANOVA and Tukey’s post hoc test. Different letters above the column indicate significant differences (*p* < 0.05).

The phytoremediation potential of pot-grown SbEXPA11-overexpressing and WT sorghum was evaluated under controlled conditions. Cd stress (10 mg/kg Cd) inhibited the growth of all experimental plants compared to untreated plants. However, the transgenic plants displayed improved tolerance, with greener and more vigorous leaves (Figure 6a). At the same time, the overexpression of *SbEXPA11* resulted in increased growth under Cd stress, as evidenced by a significant increase in biomass accumulation (Figure 6a,b). The transgenic plants also accumulated a significantly higher concentration of Cd in root and shoot tissues than WT plants (Figure 6c). We subsequently analysed soil Cd residues to evaluate the phytoremediation potential of transgenic sorghum. Transgenic sorghum plants were more effective in reducing the Cd content of soil (8.62 mg/kg) than WT sorghum plants (9.51 mg/kg) (Figure 6d). These findings suggest that *SbEXPA11*-overexpressing sorghum may be a practical option for the bioremediation of Cd-contaminated soils.

### 2.7. Overexpression of SbEXPA11 Rescues Photosynthetic Pigments and Efficiency, Stomatal Conductance, and Biomass Production

**Figure 7:**
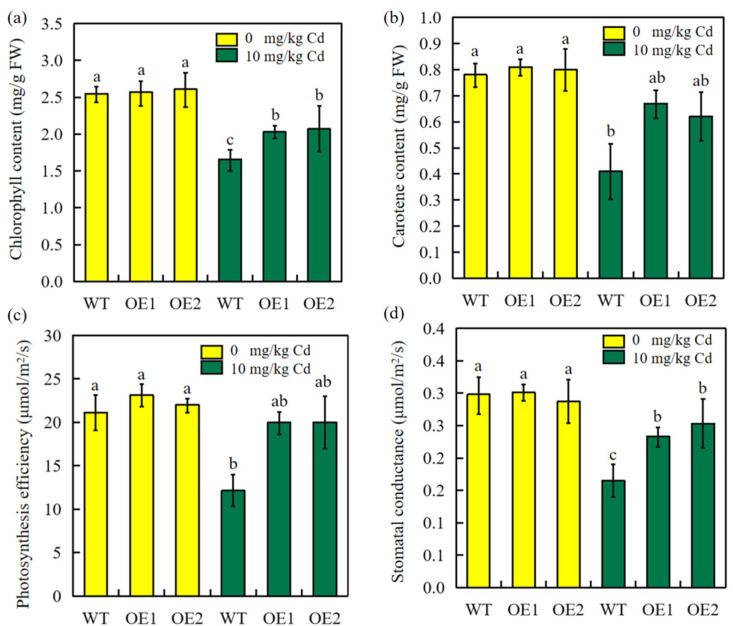
Overexpression of *SbEXPA11* alert pigment and photosynthesis efficiency under cadmium stress conditions. (**a**) Chlorophyll content. (**b**) Carotenoid content. (**c**) Photosynthesis efficiency. (**d**) Stomatal conductance. Error bars are ±SE of *n* = 3. Significant differences analysis was carried out by one-way ANOVA and Tukey’s post hoc test. Different letters above the column indicate significant differences (*p* < 0.05).

The photosynthetic pigment content and photosynthetic efficiency were evaluated 30 days after germination in pot-grown sorghum plants. The concentrations of chlorophyll and carotene were lower in stressed plants than in unstressed plants (Figure 7a,b). Notably, the transgenic plants showed much less Cd stress inhibition, and contained significantly more chlorophyll and carotene than WT plants (Figure 6a,b). Moreover, under Cd stress, transgenic plants exhibited higher photosynthetic efficiency and stomatal conductance (Figure 7c,d). Overall, transgenic sorghum is likely to exhibit better survivability under Cd-stressed conditions.

## 3. Discussion

### 3.1. The SbbHLH041 TF Activates SbEXPA11 Expression by Directly Binding to the E-Box in Its Promoter

bHLH protein family members contain the basic helix–loop–helix structural domain, which modulates downstream gene expression by specifically recognizing and binding to specific motifs (G-box/CACGTG or E-box/usually CANNTG) in the promoters of target genes [36]. For example, both AtbHLH122 and AtbHLH3 interact with the E-box cis-acting elements in the promoter of CYP707A3, thereby suppressing its activation. This regulatory mechanism enhances both osmotic and drought stress tolerance in Arabidopsis [30]. GmbHLH3 was found to interact with the G-box cis-acting element in the GmCLC1 promoter, regulating its activation in response to chlorine (Cl^−^) stress/salinity [19]. TaMYC8, a member of the bHLH superfamily, directly binds to the G-box in the TaERF6 promoter, thereby regulating the Cd stress response [37]. In this study, the SbbHLH041 protein was found to directly bind to the E-box in the P3 (−1972~−1945) fragment in the *SbEXPA11* promoter (Figure 2b,c), indicating that SbbHLH041 targets *SbEXPA11* by specifically binding to the E-box of its promoter. Furthermore, SbbHLH041 participates in a positive feedback mechanism associated with a Cd-stress-related gene regulatory network in sorghum.

### 3.2. Overexpression of SbEXPA11 Increases Cd Accumulation by Increasing Uptake and Long-Distance Transport

A growing body of evidence implicates expansin genes in the plant response to ion toxicity. For instance, overexpression of the *RhEXPA4* gene from rose leads to the remodelling of the cell wall structure, resulting in altered sodium (Na^+^) and Cl^−^ balances [38]. The transformation of *HvEXPB7* into wild barley effectively enhances K^+^ uptake [13], and the overexpression of *OsEXPA7* significantly enhances K^+^ concentrations and reduces Na^+^ concentrations in leaves and roots [39]. The poplar expansin gene *PtoEXPA12*, when overexpressed in tobacco, increases Cd uptake by 67.43% and 34.46% in roots and shoots, respectively [16]. Here, we found that after treatment with 50 μM Cd, *SbEXPA11* expression significantly increased in roots, with the maximum expression observed 12 h after exposure (Figure 1b–d). In both the hydroponics assay and pot experiment, the growth of transgenic plants was repressed by Cd exposure but was still significantly improved compared to control plants (Figure 3a and Figure 5a). Moreover, the overexpression of *SbEXPA11* significantly enhanced the aggregation of Cd in roots and shoots (Figure 3b and Figure 5c). *PtoEXPA12* promotes Cd uptake, but not transport, and effectively sequesters toxic ions [18]. Additionally, the overexpression of *TaEXPA2* increased tolerance to Cd in transgenic tobacco by limiting Cd transportation [17]. Here, transgenic sorghum overexpressing *SbEXPA11* exhibited increased Cd accumulation through the regulation of Cd uptake and long-distance transport.

### 3.3. SbEXPA11 Mediates Tolerance to Cd by Improving ROS Scavenging

Abiotic stress induces an elevation in free oxygen radicals, resulting in membrane lipid peroxidation. This process leads to the oxidative destruction of the cellular membrane system [40]. In response, plants exhibit both nonenzymatic and enzymatic mechanisms to quench excessive ROS, thereby enhancing resistance to abiotic-stress-associated damage [41]. Cytosolic enzymatic antioxidants, including CAT, POD, and SOD, detoxify ROS [42]. Here, we found that the proline content and enzymatic antioxidant activities were higher, while electrolyte leakage and MDA content were lower, in Cd-stressed transgenic plants compared to Cd-stressed WT plants (Figure 5a–c). Together, these results demonstrate that *SbEXPA11* protects sorghum from oxidative stress by improving ROS-quenching (Figure 4).

### 3.4. Overexpression of SbEXPA11 Rescues Biomass Production by Increasing Photosynthetic Efficiency

Heavy metals, including Cd, are known to inhibit photosynthesis [43]. Specifically, Cd negatively impacts plant growth through changes in stomatal conductance, respiration, transpiration, and chlorophyll biosynthesis, thus inhibiting photosynthetic activity [44]. The detrimental effects on photosynthesis induced by heavy metal stress are often accompanied by stomatal restriction [45]. The stomata manage water transport, promoting the transport of metal ions from roots to aerial tissues [46]. Our results regarding long-distance ion transport, discussed above, also confirm this notion.

Stomatal conductance dynamics are partially responsible for determining plant productivity and fitness under varied environmental conditions, serving to mediate photosynthetic efficiency [42]. Cd exposure inhibits plant growth by reducing photosynthesis and respiration [47,48,49]. Here, we found that photosynthetic efficiency was significantly inhibited under Cd stress, as were the pigment contents and stomatal conductance (Figure 7). However, transgenic plants exhibited improved photosynthetic parameters compared to control plants, indicating that the overexpression of *SbEXPA11* promotes photosynthesis under Cd exposure. Photosynthesis is the physiological basis of biomass production, of which the primary determinant is photosynthetic efficiency [50,51]. Biomass production substantially decreqased under Cd stress, although transgenic plants accumulated significantly more biomass due to their enhanced photosynthetic efficiency compared to control plants. The high biomass production of sorghum is particularly conducive to phytoremediation [52]. Therefore, we speculate that SbEXPA11 may serve as an important phytoremediation tool for contaminated soils.

### 3.5. Perspective

Phytoremediation using transgenic plants poses several challenges. Firstly, creating transgenic plants with phytoremedial potential is a difficult task. Additionally, legal restrictions on the cultivation of transgenic plants make it challenging to conduct large-scale field trials. Thus, our research project was limited to a controlled environment. At present, there are concerns regarding the potential ecosystem instability caused by the dispersion of pollen from transgenic plants. However, sorghum, an annual crop with rapid growth, provides a solution in soil remediation. In soil remediation practices, sorghum can be harvested before flowering, allowing for unified treatment, such as burning instead of using the straw for feed. This approach prevents transgenic sorghum from entering the food chain. Furthermore, conducting long-term environmental safety assessments of genetically modified crops is crucial. The safety of genetically modified crops is currently a topic of controversy, and addressing these concerns is vital in alleviating public panic surrounding genetically modified crops.

## 4. Materials and Methods

### 4.1. Plant Materials and Experimental Design

Sorghum cultivar ‘TX430’ was utilized as the experimental material in this study. For the hydroponics experiment, transgenic and wild-type (WT) seeds were germinated in darkness (26 °C) on moistened filter paper and then relocated to hydroponic tanks containing Hoagland solution, which was renewed every 2 days. Then, 3-week-old seedlings of uniform size were treated with Hoagland solution containing either 0 μM or 50 μM Cd. Samples were taken at several timepoints and processed for real-time quantitative PCR (RT–qPCR), as well as physiological and biochemical analyses.

The pot experiment was performed under greenhouse conditions using 3-week-old seedlings of uniform size. For experimental pots, 100 mg Cd was added per 10 kg of nutrient soil. Control pots contained only nutrient soil. The seedlings were watered every 10 d with 2 L Hoagland solution. Each plant was photographed 30 days after treatment.

### 4.2. RNA Extraction and RT–qPCR

An RNAprep Pure Plant Kit (Tiangen, Beijing, China) was utilized for the extraction of total RNA from the roots and shoots of seedlings. A cDNA synthesis kit (Tiangen, Beijing, China) was utilized to generate first-strand cDNA. Gene expression levels were detected with SYBR Green (Tiangen, Beijing, China) using an Applied Biosystems 7900 qPCR system. The qPCR results were normalized according to the 2−∆∆Ct method, with the expression of β-actin, which was utilized as the internal reference [53].

### 4.3. Characterization of SbEXPA11 in Sorghum

The primers used to amplify *SbEXPA11* were designed based on sequences downloaded from the Gramene database (https://ensembl.gramene.org (accessed on 15 October 2022)) (Supplementary Table S1). The *SbEXPA11* open-reading frame (ORF) was amplified from a cDNA template isolated from TX430 seedlings grown under normal growth conditions. The PCR products were then cloned into the pMD18-T vector and sequencing was conducted on a minimum of three independent positive clones (Sangon, Shanghai, China). DNAMAN ver. 6.0 (Lynnon Biosoft, San Ramon, CA, USA) was used to align the protein sequences.

### 4.4. Genetic Transformation

For the overexpression vector construction, the coding sequence (CDS) of SbEXPA11 was first amplified and then cloned into the binary vector pCambia3201 between restriction sites EcoR I and Hind III. Agrobacterium-tumefaciens-mediated transformation was carried out as described in a previously published report [54].

### 4.5. Measurement of Cd, MDA, and Proline Content, Electrolyte Leakage, ROS Content, and Antioxidant Enzyme Activities

The Cd content was determined using inductively coupled plasma–mass spectrometry (ICP–MS), as described in a previously published report [55]. The measurement of electrolyte leakage was carried out based on the methods of a previously published report [56]. The malondialdehyde (MDA) content, proline content, H_2_O_2_ content, O_2_^−^ content, and enzymatic antioxidant activity, including peroxidase (POD), catalase (CAT), and superoxide dismutase (SOD), were determined with Jiancheng Bioengineering Institute (Nanjing, China) kits.

### 4.6. Measurement of Chlorophyll and Carotene Content

The contents of carotenoid and chlorophyll of the third leaves of each sorghum plant were evaluated using a SPAD-502 PLUS chlorophyll meter (Konika Minolta, Tokyo, Japan), following a method described in a previously published report [57].

### 4.7. Measurement of Photosynthetic Efficiency, Stomatal Conductance, and Biomass Production

Photosynthetic efficiency and biomass production were measured in pot-grown sorghum plants at the point of flower initiation stage 60 days after sowing. The photosynthetic efficiency and stomatal conductance of fully expanded penultimate leaves were evaluated with a portable open-flow LI-6400 gas-exchange device (LICOR Biosciences, Lincoln, NE, USA). All measurements were conducted the morning prior to harvest, as described in a previously published report [58]. The plants were harvested and washed, and subsequently dried at 60 °C for 72 h prior to weighing.

### 4.8. Yeast One-Hybrid (Y1H) Assay

The SbEXPA11-promoter oligonucleotides were cloned into the pHIS2 yeast expression vector to serve as the reporter construct (pHIS2–SbEXPA11). The ORF of SbbHLH041 was cloned into the pGADT7 vector as the effector (pGADT7–SbbHLH041). Both the effector and reporter construct were co-transformed into ‘Y187’ yeast. Yeast cell culture was performed as described in a previously published report [59].

### 4.9. Electrophoretic Mobility Shift Assay (EMSA)

The CDS of *SbbHLH041* were cloned into the pMALc-5G vector. Subsequently, ‘DH5α’ Escherichia coli was utilized to express both the MAP protein and MBP-SbbHLH041 recombinant fusion protein. Amylose resin (New England Biolabs, Ipswich, MA, USA) was utilized for the purification of fusion proteins, utilizing the manufacturer’s standard directions. An *SbEXPA11*-promoter fragment containing an SbbHLH041 binding site was utilized as a probe, along with putative mutated binding site probes and unlabelled WT hot/cold probes as competitors. The EMSA procedure was conducted according to a previously published report [60].

### 4.10. Statistical Analyses

Statistically significant differences among results were evaluated with one-way ANOVA and Tukey’s post hoc test (*p* < 0.05). All data are shown as the means ± standard errors (SE) of three replicates.

### 4.11. Primers

All primers are listed in Supplementary Table S1.

## 5. Conclusions

The overexpression of *SbEXPA11* in sorghum plants led to an increase in Cd accumulation and a decrease in Cd toxicity, and rescue biomass production by enhancing photosynthetic efficiency. When exposed to Cd stress, the WT lines exhibited higher levels of H_2_O_2_ and O_2_^−^ compared to the transgenic plants. The transcription of *SbEXPA11* was activated by the transcription factor SbbHLH041, which binds to the E-box cis-acting element in the promoter of its target gene. Our findings suggest that the SbbHLH041–*SbEXPA11* cascade module is responsible for conferring Cd tolerance in sorghum. Additionally, the overexpression of *SbEXPA11* enhances the potential of sorghum for the bioremediation of Cd-polluted soils. Overall, this study provides a theoretical foundation for the enhancment of plant characteristics through *SbEXPA11* expression, thereby increasing the potential of sorghum plants for remediating Cd-contaminated soil.

## Data Availability

Not applicable.

## Supplementary Materials

The following supporting information can be downloaded at: https://www.mdpi.com/article/10.3390/ijms241713061/s1.
