# Supplementary material for: The SbbHLH041–SbEXPA11 Module Enhances Cadmium Accumulation and Rescues Biomass by Increasing Photosynthetic Efficiency in Sorghum
Source: Int J Mol Sci. 2023 Aug 22;24(17):13061. doi: 10.3390/ijms241713061 (PMC10487693; doi:10.3390/ijms241713061)
Supplement: Supplementary file 1 [file ijms-24-13061-s001.zip › ijms-2573650-supplementary.pdf]

Supplementary figure legends

Figure S1. PCR identification and expression level of SbEXPA11 transgenic plants

(a) Identification of transgenic sorghum lines using PCR. (b) The relative expression of *SbEXPA11* in transgenic and WT plants. Different letters above the column indicate significant differences ($p < 0.05$).

Figure S1

(a)

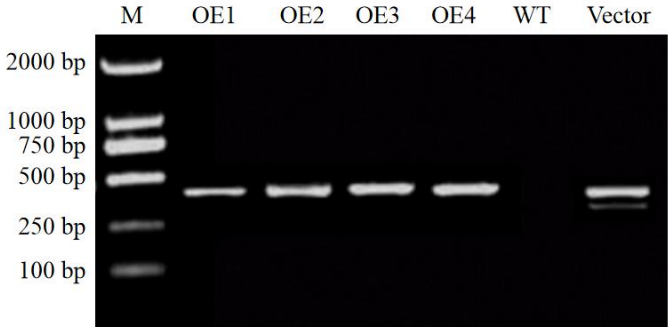

(b)

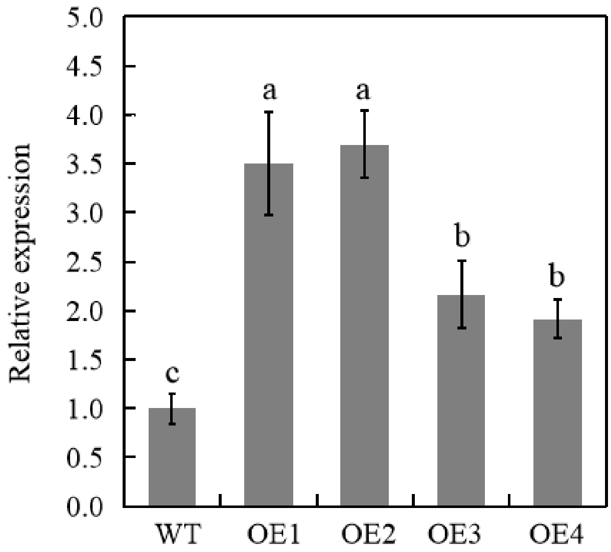

Supplementary Table S1. Primers used in this work

Primer name	Primer sequence (5'→3')	Purpose
1-F	ATGCGATTCGACCGAGG	Cloning the coding sequence of *SbbHLH041*
1-R	TCACACACCTGTCCCAG	
2-F	CATTCGAATGCAATCCACCG	qPCR for *SbbHLH041*
2-R	TCGTCGGATCGAATCGGATCAC	
3-F	ATGCGATTCGACCGAGG	Cloning the coding sequence of *SbEXPA11*
3-R	TCACACACCTGTCCCAG	
4-F	CATTCGAATGCAATCCACCG	qPCR for *SbEXPA11*
4-R	TCGTCGGATCGAATCGGATCAC	
5-F	GGTACCCACGGGCAACTAGAGTAATCC	Construction of plant expression vector. Restriction sites are underlined.
5-R	GGATCCATTTGTCCTTTTTGATATGAAA	
